# A cross-sectional assessment of population demographics, HIV risks and human rights contexts among men who have sex with men in Lesotho

**DOI:** 10.1186/1758-2652-14-36

**Published:** 2011-07-04

**Authors:** Stefan Baral, Darrin Adams, Judith Lebona, Bafokeng Kaibe, Puleng Letsie, Relebohile Tshehlo, Andrea Wirtz, Chris Beyrer

**Affiliations:** 1 Johns Hopkins Bloomberg School of Public Health, Center for Public Health and Human Rights, Epidemiology, Baltimore, USA; 2 The Matrix Support Group, Maseru, Lesotho; 3 United States Peace Corps, Maseru, Lesotho; 4 UNDP, HIV/AIDS Practice, Maseru, Lesotho; 5 National AIDS Commission, Policy, Strategy and Communications Directorate, Maseru, Lesotho

## Abstract

**Background:**

Evidence is increasing of high HIV risks among southern African men who have sex with men (MSM). This represents the first study of HIV risks and human rights contexts among MSM in Lesotho.

**Methods:**

Two hundred and fifty-two men who reported ever having anal sex with another man were accrued with snowball sampling and were administered a structured quantitative instrument in October and November 2009.

**Results:**

Of the participants, 96.4% (240/249) were ethnic Basotho with a mean age of 26.3 years (range 18-56), 49.6% (124/250) were currently employed, and 95.2% (238/250) had at least a secondary-level education. Self-reported HIV prevalence was 11.6% (22/190); 54.5% (128/235) reported being tested for HIV in the last year. HIV knowledge was low; only 3.7% (8/212) of MSM knew that receptive anal intercourse was the highest risk for HIV and that a water-based lubricant was most appropriate to use with condoms.

Bivariate associations of wearing condoms during last intercourse with men include: having easy access to condoms (OR 3.1, 95% CI 1.2-8.5, p < 0.05); being older than 26 years (OR 2.3, 95% CI 1.3-4.2, p < 0.01); knowing that receptive anal intercourse is higher risk than insertive anal intercourse (OR 2.6, 95% CI 1.2-5.9, p < 0.05); wearing condoms with female sexual partners (OR 3.5, 95% 1.4-8.3, p < 0.01); using water-based lubricants (OR 2.8, 95% CI 1.4-5.5, p < 0.01); being less likely to report having been diagnosed with a sexually transmitted infecton (OR 0.21, 95% CI 0.06-0.76, p < 0.05); and being more likely to have been tested for HIV in the last year (OR 2.0, 95% CI 1.2-3.6, p > 0.05).

Human rights abuses were common: 76.2% (170/223) reported at least one abuse, including rape (9.8%, 22/225), blackmail (21.3%, 47/221), fear of seeking healthcare (22.2%, 49/221), police discrimination (16.4%, 36/219), verbal or physical harassment (59.8%, 140/234), or having been beaten (18.9%, 43/228).

**Conclusions:**

MSM in Lesotho are at high risk for HIV infection and human rights abuses. Evidence-based and rights-affirming HIV prevention programmes supporting the needs of MSM should be developed and implemented.

## Background

Across the African continent, there has been increasing recognition of the heightened risk of men who have sex with men (MSM) to HIV infection [1-3]. HIV prevalence studies have now been completed in numerous countries of southern and eastern Africa, including South Africa, Malawi, Namibia, Botswana, Tanzania, Uganda and Kenya, and are ongoing in other countries [4,5]. Incidence data are available from Kenya [6,7]. A recent study evaluating HIV prevalence, associations with HIV infection, and human rights contexts among MSM in Malawi, Namibia and Botswana demonstrated elevated risk of HIV among MSM even in the context of generalized HIV epidemics [4].

HIV is hyperendemic among adults of reproductive age in Lesotho, with a prevalence of 23.2% in 2008, the third highest in the world [8]. The HIV epidemic appears to have peaked in 1995 with an incidence of 3.6%; more recently, in 2007, HIV incidence in the general population was estimated to be 1.7%. Lesotho has a female-predominant epidemic in which women aged 15 to 30 years have two times the HIV prevalence as compared with age-matched men (21.4% vs 10.1%, respectively).

No study has included MSM in Lesotho; this lack of data was highlighted in the Lesotho Modes of Transmission Study report, which concluded that there was a lack of evidence to make conclusions about the prevalence or HIV risk among sexual minorities[8]. Furthermore, the 2006-2011 Lesotho National Strategic Plan (NSP) states that "there isn't sufficient empirical data to help determine the extent of the epidemic" among MSM. The NSP further lists developing behaviour change and condom distribution programmes targeting MSM as strategic priorities[9]. Namibia, Botswana and Malawi have generalized epidemics in which the most well-established risk factors for transmission have been high-risk heterosexual intercourse, including multiple concurrent partnerships, and vertical transmission.

Lesotho is a low-income nation that is ruled as a kingdom with a population of just over 2 million people [10]. The country is wholly surrounded by South Africa where there has been consistent evidence of the disproportionate burden of HIV among MSM. A recent study completed by Lane *et al *using respondent-driven sampling of men in Soweto recruited 378 predominately African MSM with an overall adjusted HIV prevalence of 13.2% (95% CI 12.4-13.9) in 2008 [11,12].

Studies of MSM in Africa that have assessed structural barriers to HIV services have demonstrated widespread stigma in the form of violence, exclusion and denial of healthcare services, and targeted discrimination. In a reanalysis of the data describing MSM from Malawi, Namibia and Botswana, MSM commonly reported experienced and perceived stigma as limiting coverage and uptake of preventive services, such as HIV testing [13].

In Senegal, a qualitative case study was completed to assess the impact of the enforcement of laws that criminalize same-sex practices on HIV prevention efforts among MSM in Senegal [14,15]. After the International Conference on AIDS and STIs in Africa meeting held in Dakar, Senegal, in 2008, nine MSM health providers were arrested in the privacy of their homes as a result of their sexual orientation [16,17]. The aftermath of these arrests were studied, and respondents reported pervasive fear and hiding among MSM secondary to these arrests and subsequent media publicity [16]. Moreover, service providers suspended HIV prevention programmes targeting MSM out of fear for their own safety. Those who continued to provide services noticed a sharp decline in MSM participation. Overall, the coverage of well-established HIV prevention, treatment and care services significantly decreased in response to these arrests, in part, due to the inability of MSM to continue to advocate for their needs because of fear for their safety [14]. These data suggest that laws criminalizing same-sex practices and the enforcement of these laws create a context where it is exceedingly difficult to accrue and retain MSM secondary to the realistic fear of adverse consequences of inadvertent disclosure of sexuality.

While the epidemiology of HIV prevalence and risk among MSM across the continent is becoming increasingly clear, interventions specifically targeting MSM have lagged [18,19]. The components of an optimal package of interventions for MSM in Africa have not been studied. The first study prevention study including MSM completed in Africa was iPrEX, which evaluated oral Truvada as chemoprophylaxis for the prevention of HIV infection [20]. While this was the first study to demonstrate effectiveness of a biomedical intervention among MSM specifically, it also demonstrated the feasibility of conducting randomized controlled trials among MSM in South Africa. Other biomedical interventions, such as tenofovir-loaded rectal microbicides, are also being considered for MSM in the African context.

Baseline assessments of risk status, including bisexual concurrency, condom and lubricant use, and rates of transactional sex among MSM, are needed to consider the feasibility of biomedical interventions, such as oral and topical chemoprophylaxis. In addition, characterization of structural barriers limiting access to HIV prevention, treatment and care services for MSM are needed to inform combination HIV prevention intervention development and evaluation strategies. In response to these data needs, this study represents the first data characterizing sociodemographic characteristics, HIV-related risk practices and structural barriers to care by MSM in Lesotho.

## Methods

### Study population

Participants eligible for this research were men aged 18 years or older, who reported ever having had insertive or receptive anal intercourse with another man. Other inclusion criteria included the ability to provide informed consent in either Sesotho or English to partake in filling in the questionnaire. Exclusion criteria included being born as a female. Known positive HIV serostatus or self-identified sexual orientation were not exclusion criteria for this study.

### Sampling and recruitment

This study used snowball sampling for participant accrual. During the planning phase for this work, several different accrual strategies were discussed, including time-location sampling as compared with chain-referral methods, such as snowball or respondent-driven sampling. Given the lack of identified venues, the ability to use time-location sampling was limited as the only option was to potentially accrue from healthcare settings. There are no dedicated service providers for MSM in Lesotho. Based on safety and desired study outcomes, the decision was made to use peer-referral methods.

The option of training to complete respondent-driven sampling was presented to the community implementing partner, the Matrix Support Group, but a decision was made that, based on existing infrastructure, the implementation of respondent-driven sampling would be a barrier. The snowball sample was initiated with 10 seeds chosen from the membership of the Matrix Support Group, which is the first and only organization supporting the health and advocacy needs of lesbian, gay, bisexual and transgendered (LGBT) peoples in Lesotho http://www.facebook.com/pages/Matrix-Support-Group/294891844889.

These seeds also functioned as the study interviewers and were chosen based on their high level of motivation for this research project and being articulate in their explanation of the purpose and expectations of the study. Moreover, the seeds were chosen to maximize diversity with respect to sociodemographics and known risk practices. These participants were provided with a two-day training session in a central location covering the purpose of the research, design of the research project, human subjects' protection, interviewing methods, the prevention of duplicate study participation, and data integrity. The seed participants completed the study questionnaires before the beginning of the two-day training session to minimize any bias associated with the training.

The 10 seeds recruited social network contacts meeting inclusion criteria for interviews, and then provided training to those contacts about how to recruit others until the desired sample size was achieved. All initial seeds accrued a maximum of 10 people each and the sample size was achieved within three weeks or approximately three waves of recruitment. Study interviews were completed in three major urban centres across Lesotho and study interviewers were given a certificate of research training at the end of the research project. Each study participant received the equivalent of US$5 for their participation, which was determined based on the estimated cost of return transportation and a meal.

### Community mobilization and results validation

This was the first study to attempt to accrue MSM in Lesotho. To sensitize the LGBT community, a series of community engagement sessions were planned in partnership with the Matrix Support Group. These community engagement sessions were completed in rented venues with all communication about the meetings done through existing social and sexual networks. In total, eight community engagement sessions were completed with total attendance of approximately 400 men and women. These sessions provided a forum for discussion of the purpose of the research and getting feedback on study design and implementation plans.

It was at these sessions that decisions regarding accrual strategies and optimal timing were made, resulting in the use of snowball sampling. However, no actual study accrual happened at these events. Leadership of the Matrix Support Group was included at all stages of the research project for consultation and guidance. After the preliminary analysis was complete, the results were presented to the community leadership for validation.

### Sample size calculation

As there was no biologic or objective outcome of this study, the sample size was determined based on self-reported condom use. Specifically, the study was powered on the assumption that those who had received information about preventing HIV infection from other men would have a 15% increase in reported consistent condom use. There was no previous estimate of condom use among MSM in Lesotho; however, using our own data from Malawi, Namibia and Botswana, it was assumed that a conservative estimate of regular condom use during anal sex with other men among MSM was 50%.

Furthermore, a systematic review and meta-analysis of the literature on behavioural interventions targeting MSM has demonstrated that interventions can increase reported condom use by approximately 16.5% in all risk categories of MSM [18,19]. A power analysis demonstrated that for 80% power, we would require 165 participants. For respondent-driven sampling, design effect has been assumed to be at least 1.5. We used an assumption of a design effect of 1.5 and estimated a necessary sample size of 250 to achieve a type I error probability of 0.05 associated with the test of the null hypothesis.

### Study instrument and interviews

A short, structured survey instrument containing 45 questions was developed in consultation with a variety of key informants in Lesotho, including community members, the National AIDS Commission, the Ministry of Health, and the Joint United Nations Programme on HIV/AIDS (UNAIDS) and the United Nations Development Programme (UNDP). Domains of this instrument include baseline demographics, relationship patterns, sexual practices, HIV risk status, health-seeking practices and human rights contexts. The instrument was based on a survey instrument that has been used widely by our group among MSM in southern Africa, although locally adapted by piloting with leaders of the Matrix Support Group. Interviews were completed by trained interviewers derived from the MSM community, took approximately 25-30 minutes to complete, and collected no identifiable information.

### Statistical analysis

Data were doubly entered into Microsoft Excel at the Johns Hopkins School of Public Health and in Lesotho and subsequently imported to Stata 10.0 for analysis. Bivariate analyses included two-sample tests for differences in proportions, χ^2 ^tests of independence, and bivariate logistic regression assessing the relationship between risk factors and HIV risk status. Variables that are significantly (p < 0.05) associated with the outcome of interest are reported by presenting adjusted odds ratios (ORs) with 95% confidence intervals (CIs) and associated p values. Participants were freely able to decline answering certain questions and were reminded of this during the informed consent process. Consequently, there are varying denominators observed throughout the Results section based on the total number of study participants who answered any one particular question in the survey.

### Ethical approval and funding

Ethical approval was sought and received from the Ministry of Health and Social Welfare in Lesotho and the Johns Hopkins School of Public Health. This study was funded as a joint initiative between UNAIDS and UNDP in Lesotho.

## Results

The 252 study participants had a mean age of 26.3 years with a range of 18 to 56 years. Study participants were mostly ethnic Basotho (96.4%, 240/249), approximately half were currently employed (49.6%, 124/250), and 59.2% had a tertiary or vocational-level education (Table 1). Nearly three-quarters of participants reported being of urban origin, defined as being from cities with a population of greater than 100,000 people (72.6%, 172/237). When asked about sexual orientation, 58.6% (137/234) self-reported as being gay, 26.5% (62/234) self-reported as bisexual, and 13.7% (32/234) self-reported as heterosexual. Seventy-six of 234 participants (32.5%) had disclosed the fact that they engage in same-sex practices with other men to any member of their immediate or extended family, whereas 24.4% (56/230) had disclosed these sexual practices to even a single healthcare worker. In total, 25.2% (56/222) reported ever being married, including 16.7% (37/222) who were currently married, 6.3% (14/222) who were divorced, and 2.3% (5/222) who were widowed. Forty-four of 207 (21.3%) reported having children, all of whom reported ever having been married.

**Table 1:** Selected characteristics of men who have sex with men in Lesotho

Characteristic		Proportion (n)
Age	Mean (range)	26.3, (18-56)

	18-25 years	55.6% (124/223)

	26 and older	44.4% (99/223)

Ethnic group	Basotho	96.4% (240/249)

	White	1.2% (3/249)

	Asian/Indian	0.4% (1/249)

	Other African descent	2.0% (5/249)

Currently employed		49.6% (124/250)

City dweller		72.6% (172/237)

Education	Secondary or less	40.8% (102/250)

	Tertiary or more	59.2% (148/250)

Self-reported sexual orientation	Gay/homosexual	58.6% (137/234)

	Bisexual	26.5% (62/234)

	Heterosexual/straight	13.7% (32/234)

	Transgender	1.3% (3/234)

Disclosed sexual behaviour to a family member		32.5% (76/234)

Disclosed sexual behaviour to a health care worker		24.4% (56/230)

Married at any point in life		25.2% (56/222)

Have children		21.3% (44/207)

Number of MSM who are personally known, mean/median (range)		65/30 (0-900)

Number of known MSM that were seen or talked to in the last 6 months, mean/median (range)		37/18 (0-767)

There was great variability in the estimates of network sizes of MSM with a mean of 65, median of 30 and a range of 0-900. There was similar variability among the number of those seen in the last six months with a mean of 37, a median of 18 and a range of 0-767. Significantly more men had received any information from health providers regarding how to prevent HIV infection from women as compared with preventing infection from men (76.8% vs 63.3%, z = 3.97, p < 0.001). HIV-related information among participants was limited, with 19.0% (47/248) reporting that anal sex was the highest risk sexual practice for HIV transmission.

Moreover, 58.9% (146/248) reported that vaginal, anal and oral sex all carried the equivalent probability of HIV acquisition and transmission; 16.3% (40/245) correctly reported that receptive anal intercourse carried higher per coital act risk than insertive anal intercourse. Finally, less than half of men (44.8%, 98/219) knew that a water-based lubricant was the safest to use for anal intercourse. More than 30% of men (30.6%, 67/219) reported that petroleum-based products were safest to use. Only 3.8% (8/212) of participants gave the correct answers to these three questions regarding sexual transmission.

Fourteen study participants (7.7%, 14/183) reported injecting illicit drugs in the last 12 months, with 30.8% (76/247) of all study participants reporting being unaware that HIV could be transmitted parenterally. Alcohol use was common, with 33.8% (75/222) reported drinking alcohol more than five days per month; nearly half of the sample (47.8%, 110/230) reported being less likely to use condoms during sexual intercourse when drunk. In all, 22.2% (54/243) reported having received money or goods for anal sex with men, and 27.7% (65/235) reported having paid money or goods for anal sex with men; 35.9% (87/242) reported either paying or receiving money or goods for sex.

Table 2 shows key sexual practices among MSM in Lesotho. The majority of men (75.1%, 172/229) reported having a regular male partner; 44.3% (94/212) reported having a regular female partner; 28.6% (58/203) reported concurrent regular male and female partners; and 41% (71/173) reported active bisexuality with both male and female sexual partners in the last year. Fifty-three of 222 (28.4%) reported five or more male sexual partners in the last year, and 20.2% (38/188) reported three or more female partners during the same time frame. Condom use during last sexual encounter with a regular male partner was reported by 62.5% (135/216) of men, and 59.8% (128/214) reported condom during last sex with a non-regular male partner. With women, 32.8% (40/122) reported wearing a condom during last sexual intercourse, which was significantly less than condom use during last sexual intercourse with any male partner (45.5%, 94/207, p < 0.01). Self-reported HIV prevalence was 11.6% (22/190), with 54.5% (128/235) of men reporting being tested for HIV in the last year; 15.4% (35/228) reported having clinical symptoms consistent with a sexually transmitted infection (STI) in the last year, whereas 8.1% (19/235) reported having been diagnosed with an STI at a clinic in the last year.

**Table 2:** HIV sexual risk practices among MSM in Lesotho

Characteristic		Proportion (n)
Have regular male partner		75.1% (172/229)

Have regular female partner		44.3% (94/212)

In last 6 months:	Concurrency of *both *male and female partners	28.6% (58/203)

	Active bisexuality (not with a regular partner)	41.0% (71/173)

Number of male partners in last 12 months	Less than 5	71.6% (159/222)

	5 or greater	28.4% (63/222)

Number of female partners in last 12 months	Less than 3	79.8% (150/188)

	3 or greater	20.2% (38/188)

Condom at last sex with	Regular male partner	62.5% (135/216)

	Non-regular male partner	59.8% (128/214)

	Any male partner	45.4% (94/207)

	Any female partner	32.8% (40/122)

Bivariate associations of wearing condoms during last intercourse with men include: having easy access to condoms (OR 3.1, 95% CI 1.2-8.5, p < 0.05); being older than 26 (OR 2.3, 95% CI 1.3-4.2, p < 0.01); knowing that receptive anal intercourse is higher risk than insertive anal intercourse (OR 2.6, 95% CI 1.2-5.9, p < 0.05); wearing condoms with female sexual partners (OR 3.5, 95% 1.4-8.3, p < 0.01); using water-based lubricants (OR 2.8, 95% CI 1.4-5.5, p < 0.01); being less likely to report having been diagnosed with a sexually transmitted infection (OR 0.21, 95% CI 0.06-0.76, p < 0.05); being more likely to have been tested for HIV in the last year (OR 2.0, 95% CI 1.2-3.6, p > 0.05); and being less likely to report having been afraid to seek healthcare (OR 0.40, 95% CI 0.20-0.82, p < 0.05). Self-reported HIV infection was significantly associated with: reporting transactional sex (OR 3.1, 95%CI 1.2-7.7, p < 0.05); having more than five male partners in the last year (OR 2.6, 95% CI 1.0-6.7, p < 0.05); having been diagnosed with an STI (OR 8.6, 95% CI 2.8-26.6, p < 0.01); having had symptoms of an STI in the last year (OR 8.2, 95% CI 2.9-23.6, p < 0.01); having been raped (OR 3.4, 95% CI 1.1-10.7, p < 0.05); and partaking in injecting drug use in the last year (OR 5.1, 95% CI 1.1-22.5, p < 0.05).

Table 3 demonstrates that human rights abuses were common among MSM in Lesotho, with 76.2% (170/223) reporting at least one abuse related to their sexuality. Rights abuses include rape (9.8%, 22/225), blackmail (21.3%, 47/221), fear of seeking healthcare (22.2%, 49/221), police discrimination (16.4%, 36/219), verbal or physical harassment (59.8%, 140/234), or having been beaten (18.9%, 43/228). In addition, 9.2% (19/207) reported that their first sexual encounter with a man was with a member of their own family. In bivariate analyses, blackmail was significantly associated with having disclosed sexual orientation to a healthcare worker (OR 3.6, 95% CI 1.8-7.3, p < 0.05) or family member (OR 2.7, 95% CI 1.4-5.3, p < 0.05).

**Table 3:** The prevalence of human rights abuses among MSM in Lesotho

Characteristic		Proportion (n)
Have ever been raped		9.8% (22/225)

As a result of sexual orientation or practice, in last 3 years:	Lost employment	4.8% (10/208)

	Afraid to seek healthcare services	22.2% (49/221)

	Denied healthcare services	3.2% (7/218)

	Felt healthcare workers unable to meet unique needs	17.7% (38/215)

	Heard healthcare workers gossiping	16.1% (35/217)

	Felt legal or police discrimination	16.4% (36/219)

	Been verbally or physically harassed	59.8% (140/234)

	Blackmailed	21.3% (47/221)

	Beaten up	18.9% (43/228)

*"Yes" to any of the above human rights abuses related to sexuality*		76.2% (170/223)

## Discussion

The primary outcomes of this study included demonstrating high levels of self-reported unprotected anal intercourse, low usage rates of water-based lubricants during anal sex, and sub-optimal access to appropriate and safe healthcare services. While it would have been ideal from an epidemiological perspective to include biological outcomes, such as HIV and syphilis, the LGBT community did not consider this to be a priority and wanted to focus on a behavioural survey. Given that the per-coital probability of HIV transmission from unprotected receptive anal intercourse is approximately of 0.82% (95% CI 0.24, 2.76)[21], this sexual practice represents an excellent predictor of HIV infection. Furthermore, self-reported HIV prevalence was used as an outcome and was found to be 11.6% among this relatively young sample of men in Lesotho.

Earlier studies have indicated that self-reported HIV prevalence rates tend to significantly underestimate true HIV prevalence among MSM in Africa with as few as one in 20 MSM correctly reporting positive HIV serostatus [4,22]. The reasons for underreporting are multifactorial and include participants not knowing their HIV status or being unwilling to disclose their status in the context of a research study. HIV prevalence is approximately 10.1% among men aged 15 to 30 years in the general population of Lesotho, indicating that the men sampled here likely carry a significant disproportionate burden of HIV [8].

There are several limitations to this cross-sectional study. Resources and the constraints of working with small community-based organizations in difficult social environments limited the scale and scope of this study. While working with these organizations can be difficult, there are several important reasons why this is a crucial component of this assessment. Primarily, research teams have limited access to the community of MSM without effective engagement and empowerment of local community groups. In addition, involvement and ownership in these studies builds capacity for further projects to be completed.

However, the initial seeds for this study also acted as interviewers for this project. While they were interviewed before receiving interviewer-related training, this likely introduced significant selection bias limiting external validity of these results. We contend that the results indicating condom usage levels is likely to be a conservative estimate of risk among MSM in Lesotho as study participants have overlapping social and sexual networks with MSM that are involved in the only support group and service provider for MSM in Lesotho. This same selection bias likely overestimated the actual number of MSM who would self-report as gay or homosexual among all MSM in Lesotho.

Due to the cross-sectional nature of this study, directions of causality could not be established. The study sample was a convenience sample generated by use of chain-referral techniques rather than population-based samples, which is a key limitation with this study methodology, limiting the generalizability of the results to the wider population of MSM in Lesotho. While interviewers received two days of training related to interviewing skills, data quality could still be an issue related to social desirability biases and incorrect coding of surveys. This bias was minimized by education of the interviewers and piloting of the instruments in Lesotho. The interviews were completed in urban centres, further limiting the generalizability of these results.

While this study had numerous limitations, as mentioned, it draws strength from the fact that this community-led research project was able to quickly accrue MSM. In fact, accrual was completed within three weeks, and was terminated because the study sample size had been reached although there were many more people who wanted to partake in this project. Moreover, this study was the first to demonstrate the risk status of MSM in Lesotho and was completed with significant guidance and leadership from Lesotho's only LGBT community-based organization, the Matrix Support Group. The process and results indicate that MSM exist in Lesotho who report high-risk HIV practices and numerous structural barriers to accessing care.

As previously mentioned, Lesotho has a highly generalized HIV epidemic, although new infections in the general population appear to have decreased throughout the last decade [9]. HIV prevention efforts should and do reflect this generalized epidemic with the focus on decreased high-risk heterosexual transmission with a package of services, including behavioural components focused on increasing condom use and partner reduction, biomedical components moving towards the scale up of male circumcision, and increased coverage of antiviral programmes and structural programmes focused on gender equality and other key structural barriers [8]. However, the majority of these interventions do not address risk associated with same-sex practices.

This study demonstrated suboptimal HIV knowledge in terms of risks associated with receptive anal intercourse, low rates of condom use during anal sex, and inadequate use of water-based lubricants. Interventions, including peer education and the targeted distribution of condoms and water-based lubricants, would likely mitigate some of these well-established risk factors for HIV [18,19]. Moreover, bisexual practices and bisexual concurrency were found to be relatively common in this study of MSM in Lesotho, similar to what has been observed in other studies of MSM in Africa [23].

Bisexual practices among MSM in Lesotho highlight that these sexual networks are not closed and must be addressed as part of a comprehensive HIV strategy, even in the context of a generalized epidemic. There are numerous programmes focused on addressing multiple concurrent partnerships in the southern African context, though none of these programmes, as known to the authors, currently includes modules on the special needs of those practicing bisexual concurrency [24]. The results of this study highlighted a number of human rights violations, including physical and violent assault based on sexuality, representing the structural barriers to accessing HIV prevention, treatment and care services. Moreover, men reported perceived stigma in healthcare settings concurrent with observations among other African MSM [25]. These data, taken together, point to the existence of a population at high risk for HIV in Lesotho, and a population whose prevention needs are not met by existing programmes.

## Conclusions

There has been an emergence of new biomedical tools for HIV prevention for MSM, including continuous and intermittent antiviral chemoprophylaxis, lowered transmission through decreased community viral loads, and rectal microbicides [20,26,27]. The evaluation and implementation of these biomedical tools are predicated on the ability to identify MSM, to prospectively follow these men, and to ensure high levels of adherence to prevention strategies.

This study demonstrated high levels of fear in accessing healthcare, as well as blackmail, which was associated with disclosure of sexual orientation to a healthcare worker. Moreover, fear of seeking healthcare was significantly associated with lower rates of condom usage during anal sex among MSM. These structural barriers limit the ability to implement biomedical interventions, further highlighting the need for interventions for MSM to simultaneously address multiple levels of HIV risk, including at the level of the individual, community and government [28].

However, we should not wait until we have efficacious biomedical HIV prevention strategies to provide services for these men. Given low levels of knowledge observed about risks associated with receptive anal intercourse, providing systematic education about the risks associated with unprotected anal intercourse represents an effective starting point.

## Competing interests

The United Nations Development Programme (UNDP) provided financial support for this work. In addition, UNDP provided input to the design of the study instruments and encouraged the development of a report describing the work and conclusions. The UNDP played no role in the decision to publish these data in a peer-reviewed manuscript. All authors declare that they have no financial or non-financial commercial interests that may be relevant to the submitted work.

## Authors' contributions

SB participated in the design and coordination of the study, participated in data analysis, and helped draft the manuscript. DA, JL and BK carried out data collection activities, participated in the analysis and interpretation of the data, and helped draft the manuscript. PL, RT, AW and CB participated in study design and coordination, and helped revise the manuscript for intellectual content. All authors read and approved the final manuscript.
